# Charcot's Russian pupils

**DOI:** 10.1055/s-0044-1789226

**Published:** 2024-09-10

**Authors:** Hélio Afonso Ghizoni Teive, Léo Coutinho, Carlos Henrique Ferreira Camargo

**Affiliations:** 1 Universidade Federal do Paraná, Departamento de Clínica Médica, Serviço de Neurologia, Curitiba PR, Brazil.; 2 Universidade Federal do Paraná, Programa de Pós-Graduação em Medicina Interna, Disciplina de Doenças Neurodegenerativas, Curitiba PR, Brazil.

**Keywords:** History of Medicine, Neurology, Russia, Jean-Martin Charcot, História da Medicina, Neurology, Federação Russa, Jean-Martin Charcot

## Abstract

The establishment of Russian neurology in the late 19th century was significantly shaped by the neurology department at La Salpêtrière Hospital under Professor Jean-Martin Charcot's leadership. A group of Russian neurologists, guided by Professor Kozhevnikov and featuring his disciples such as Korsakov, Minor, Darkshevich, and Bekhterev, had the privilege of being mentored by Professor Charcot. Subsequently, they played pivotal roles in founding various neurology services in Russia, greatly influenced by the teachings and insights they acquired under Charcot's tutelage.

## Resumo

A criação da neurologia russa no final do século XIX foi significativamente moldada pelo departamento de neurologia do Hospital La Salpêtrière, sob a direção do Professor Jean-Martin Charcot. Um grupo de neurologistas russos, orientado pelo Professor Kozhevnikov e com discípulos como Korsakov, Minor, Darkshevich e Bekhterev, teve o privilégio de ser orientado pelo Professor Charcot. Posteriormente, desempenharam papéis fundamentais na fundação de vários serviços de neurologia na Rússia, muito influenciados pelos ensinamentos e conhecimentos que adquiriram sob a tutela de Charcot.

## INTRODUCTION

In the latter half of the 19th century, Professor Jean-Martin Charcot emerged as a global luminary in the realm of neurology. 1 2 3 Charcot's influence extended far and wide, with a multitude of French disciples and collaborators. 1 2 3 4 5 Similarly, a substantial cohort of medical practitioners from across the globe went to Charcot's neurology department at La Sultrier between 1862 and 1893 to seek training. 3 6

Among these international visitors, it is noteworthy to mention those of Russian origin. Notable individuals among them include Aleksej Kozhevnikov (1836–1902), Korsakov (1853–1900), Lazar Minor (1855–1942), Livery Darkshevich (1858–1925), and Vladimir Bekhterev (1857–1927). 6

## ALEXEJ YAKOVLEVITCH KOZHEVNIKOV

**Figure 1:**
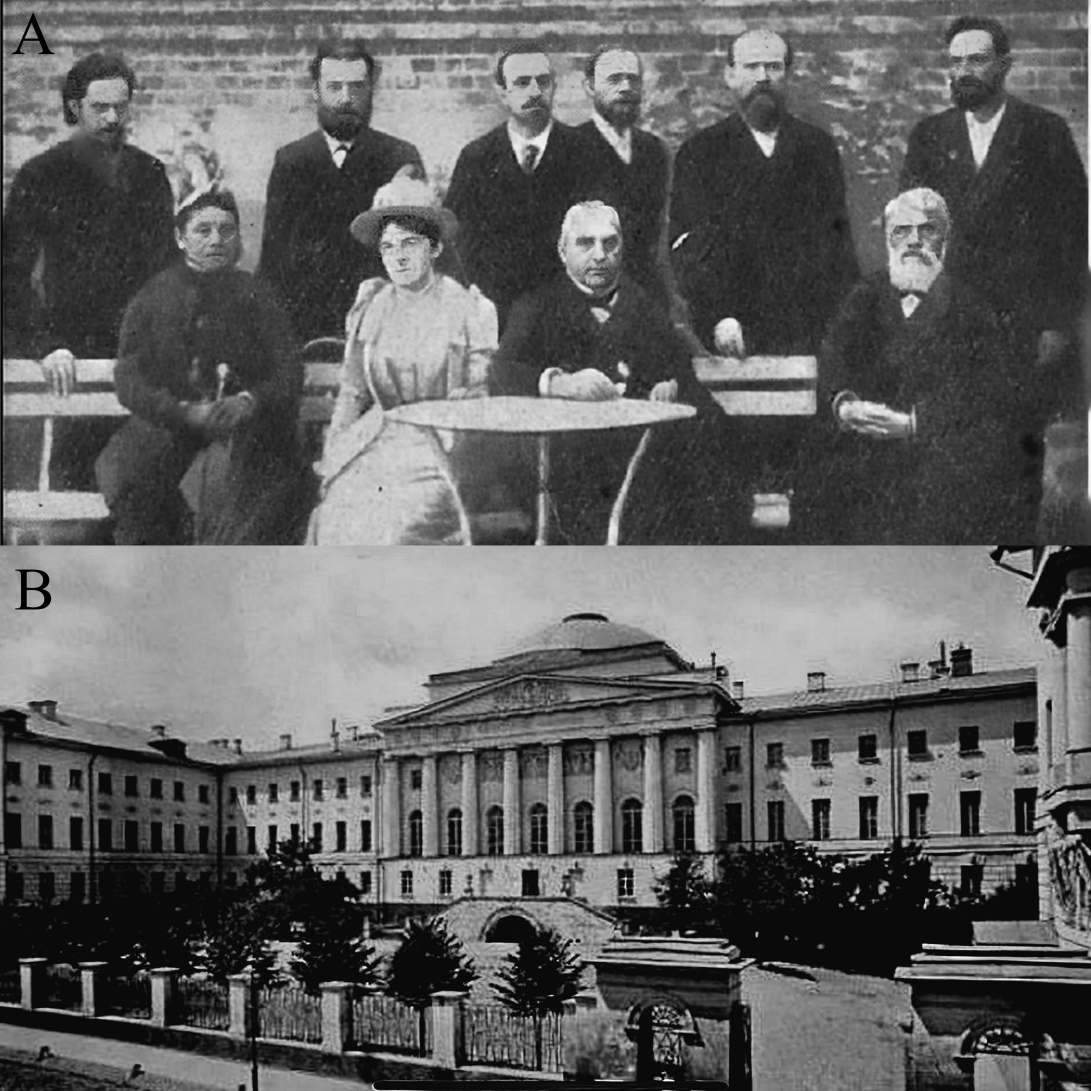
( **A** ) Moscow Imperial University in the latter XIX century. ( **B** ) Professor Charcot and his children together with Professor Kozhevnikov and his disciples in Russia in 1881. From left to right: Vladimir Muratov, Grigory Rossolimo, Jean-Baptiste Charcot, Georgii I. Pribytkov, Vladimir K. Rot, and Lazar Minor. Seated at the table are Jean-Martin Charcot and his daughter Jeanne. On the right, Aleksej Y Kozhevnikov.

Kozhevnikov was born in 1836 in the provincial city of Ryazan. He earned his medical degree from Moscow Imperial University ( Figure 1A ) and subsequently pursued internships in various countries, including Germany and the United Kingdom. 6 7 8 9 At La Salpêtrière, he worked in Charcot's neuropathology laboratory from 1867 to 1868, where he mastered the anatomical-clinical approach. 6 Upon returning to Russia, Kozhevnikov established the Moscow School of Neurology and became Russia's first professor of neuropathology. 6 7 8 9 Initially, Kozhevnikov's research aligns with Charcot's, with publications on amyotrophic lateral sclerosis, tabes dorsalis, and aphasia, eventually shifting his focus to epilepsy. 6 7 8 9 10

Kozhevnikov gained recognition for presenting four cases of *epilepsia partialis continua* at a meeting of the Moscow Neurological and Psychiatric Society in 1894. 10 Kozhevnikov passed away in 1902 due to prostate cancer. 7 Figure 1B depicts Kozhevnikov alongside his disciples during a visit of Charcot to Russia in 1881.

## SERGEI SERGEJEWITCH KORSAKOV

**Figure 2:**
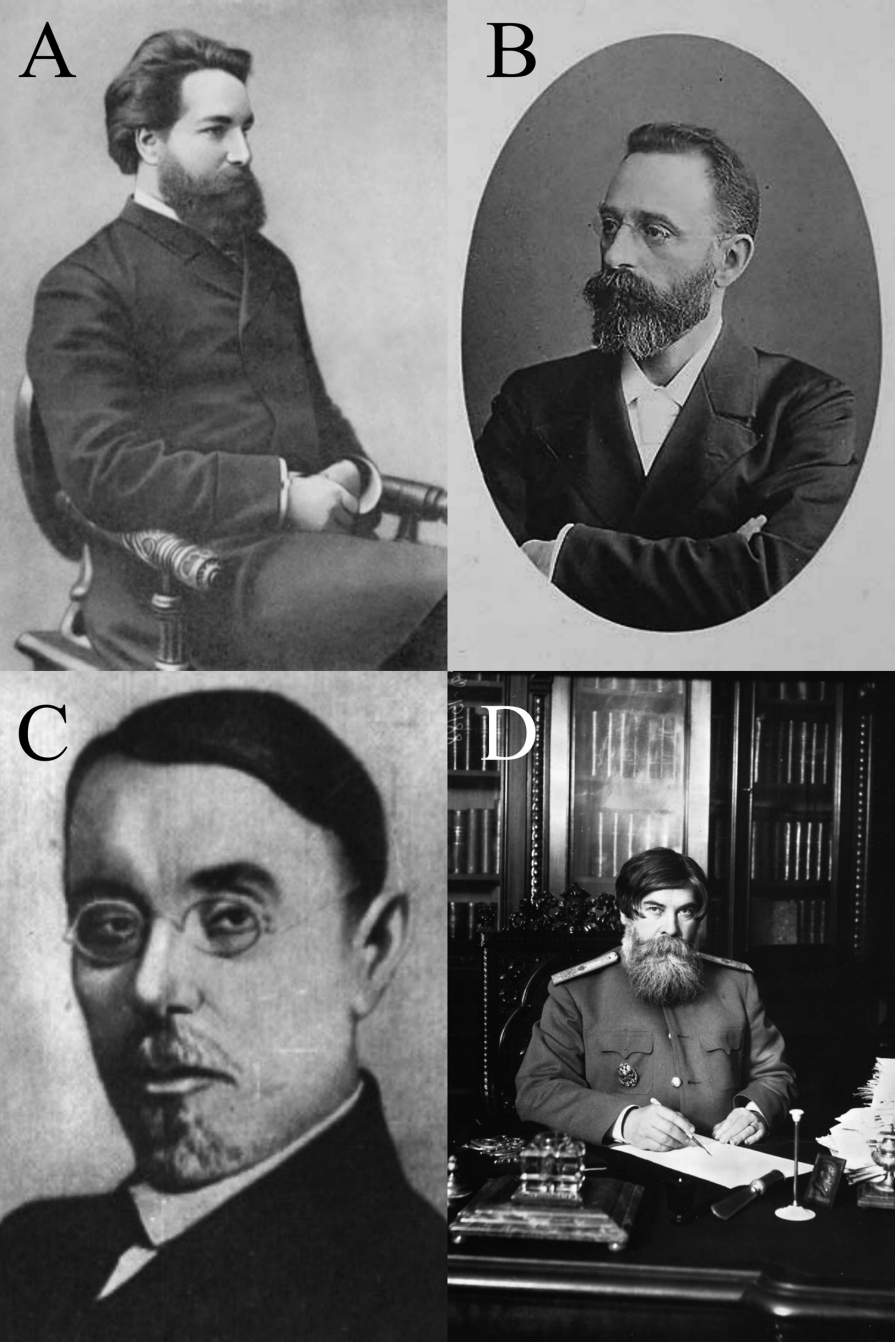
Charcot's Russian pupils. Korsakov ( Figure 2A ) was born in 1853 in a large village in central Russia. Minor ( Figure 2B ), born in Vilnius in 1855, earned his medical degree from the Moscow Imperial University. Darkshevich ( Figure 2C ), born in 1858 in Yaroslavl, completed his medical education at the Moscow Medical School. Bekhterev ( Figure 2D ), born in 1857 in Sorali, distinguished himself as an exceptional student of I. P. Merzheevskii (1838–1907).

Korsakov ( Figure 2A ) was born in 1853 in a large village in central Russia. He assumed the role of Kozhevnikov's assistant in 1876 and ventured to Paris to intern in Charcot's department. Additionally, he worked in a psychiatry department overseen by Valentin Magnan (1835-1916), a prominent figure with a keen interest in alcoholism. 6 7 8 9 11

Upon his return to Moscow, Korsakov collaborated closely with Kozhevnikov and researched polyneuropathy and alcoholism. 6 7 8 9 However, Korsakov eventually took charge of the field of mental illness, authoring several articles on the subject between 1887 and 1891. One of his earliest works, titled “Some cases of individual cerebropathy in polyneuritis ( *cerebropathia psychica toxaemica* )”, garnered significant attention. 6 Korsakov achieved worldwide recognition for this comprehensive description, which subsequently gained global prominence as the Korsakov syndrome, often associated with the Wernicke syndrome. After 2 heart attacks at the age of 44, he passed away in 1900, at the age of 46, as a result of heart failure. 6 7 8 9 11

## LAZAR SOLOMONOVICH MINOR

Minor ( Figure 2B ), born in Vilnius in 1855, earned his medical degree from the Moscow Imperial University. He was another dedicated disciple of Kozhenikov who pursued internships in various European countries, including France and Germany. 6 7 8 12 While in Paris, Minor had the privilege of working at Charcot's department. Upon returning to Moscow, he played a pivotal role in founding the Moscow Society of Neurologists and Psychiatrists, as well as the State Medical Institute of Moscow. 6 7 8 12

Minor made significant contributions to the field of neurology through his scientific publications, particularly in spinal cord trauma. He was notably recognized for describing clinical indicators that differentiate lumbago from sciatica pain, as well as elucidating the acute spinal cord hemorrhage syndrome. 6 12 However, Minor achieved international acclaim for his work on familial essential tremor. In his time, it was referred to as *status macrobioticus multiparus* , with a clinical triad encompassing tremor, longevity, and fecundity. This entity was designated as *tremor multiparus macrobioticus* of Minor and constitutes what is now recognized as essential tremor. He died in Tashkent in 1942. 6 12

## LIVERY OSIPOVICH DARKSHEVICH

Darkshevich ( Figure 2C ), born in 1858 in Yaroslavl, completed his medical education at the Moscow Medical School and was a disciple of Kozhevnikov, who supervised his thesis, titled “The conduction of light stimulus from the retina to the oculomotor nucleus”. 6 13 Between 1883 and 1887, Darkshevich undertook several internships at various European neurological centers, including Vienna, Leipzig, Berlin, and Paris. 6 7 8 In Paris, he collaborated with Charcot, utilizing the renowned anatomical-clinical method. He also worked alongside Jules Dejerine (1849–1917) and coauthored a scientific paper on muscle disorders in the tabes dorsalis. 6

Throughout his internships in Vienna and Paris, Darkshevich maintained a close friendship with Sigmund Freud (1856–1939). 6 13 Upon returning to Russia, Darkshevich coordinated the Department of Nervous Diseases at Kazan Imperial University, establishing the prestigious Kazan School of Neurologists. 6 Subsequently, he relocated to Moscow and founded the Superior State School of Moscow. Darkshevich was acclaimed for his discovery of a brainstem nucleus in the midbrain and classified as one of the accessory oculomotor nuclei. This nucleus is located near the periaqueductal gray matter and is named after him. Darkshevich died in 1925 due to a cerebrovascular disease. 6 8 13

## VLADIMIR MIKHAILOVICH BEKHTEREV

Bekhterev ( Figure 2D ), born in 1857 in Sorali, distinguished himself as an exceptional student of I. P. Merzheevskii (1838–1907), a prominent professor of psychiatry in St. Petersburg, Russia. 6 14 In 1883, after completing an internship in Leipzig, Bekhterev embarked on an internship in Charcot's neurology department in Paris. During this period, he collaborated with Pierre Janet (1859–1947), one of Charcot's associates in the psychiatry department. Bekhterev also pursued an internship with Magnan in Paris, further broadening his expertise. 6 14

With an interest in neuropsychiatry, particularly neuropsychology, Bekhterev became fascinated by Charcot's investigations into hypnosis. Upon his return to St. Petersburg, he established the Psychoneurological Institute in 1907. 6 14 Bekhterev contributed particularly to the areas of hypnosis and hysteria, publishing numerous articles. 6 He revered Charcot, whom he affectionately called the creator of modern neurology. He died suddenly while delivering a presentation, under unclear circumstances in 1927, at age 70. 6 15 16

In conclusion, Western Europe greatly influenced the origin of Russian Neurology. After touring neurological services in France, Germany, England, Austria, and other locations throughout Europe, Kozhevnikov and his pupils played a pivotal role in establishing a distinguished neurology school, leading various neurological services in Moscow and St. Petersburg. 6 8 All these accomplished individuals, including Kozhevnikov, received neurology training in Paris at the renowned neurology department of La Salpêtriére, and are rightfully regarded as pupils of Jean-Martin Charcot. 6 8 15 16 17 18
